# In Vitro Study of Blood Clot Identification and Composition Assessment by Different Magnetic Resonance Sequences

**DOI:** 10.7759/cureus.16229

**Published:** 2021-07-07

**Authors:** Yonghong Ding, Mehdi Abbasi, Yang Liu, Daying Dai, Ramanathan Kadirvel, David F Kallmes, Waleed Brinjikji

**Affiliations:** 1 Radiology, Mayo Clinic, Rochester, USA; 2 Neuroradiology, Mayo Clinic, Rochester, USA

**Keywords:** susceptibility-weighted imaging, stroke, thrombectomy, blood clot, magnetic resonance imaging

## Abstract

Background

Growing data suggest that clot composition can impact revascularization outcomes and can potentially guide treatment strategies for stroke patients with large vessel occlusion. We performed an in vitro study to determine which magnetic resonance (MR) signaling characteristics correlate with clot compositions.

Methodology

A total of 25 clot analogs were prepared by mixing human plasma and red blood cells (RBCs) with five different combinations (five samples for each combination), namely, Group A, fibrin-rich (95% plasma:5% RBCs); Group B, fibrin-rich (75% plasma:25% RBCs); Group C, intermediate (50% plasma:50% RBCs); Group D, RBC-rich (25% plasma:75% RBCs), and Group E, RBC-rich (5% plasma:95% RBCs). The prepared samples were then scanned with quantitative T2* mapping, T2 fast spin-echo (FSE), T2 gradient-echo (GRE), fluid-attenuated inversion recovery (FLAIR), and susceptibility-weighted angiography (SWAN). Thrombus-T2* relaxation time (TT2*RT) and signal intensity (SI) from different scanning sequences were measured in all groups. SIs between different groups were compared using a one-way analysis of variance. Correlation between TT2*RT and SI was determined using the Pearson correlation test.

Results

The average TT2*RT decreased from 126 ms to 37 ms from fibrin-rich to RBC-rich clots (Groups A to E). Mean SIs of Groups D and E were lower than Groups A, B, and C on T2 mapping, T2 FSE, T2 GRE, FLAIR, and SWAN images (p < 0.00001). TT2*RT and SI were positively correlated on T2 mapping (R = 0.9628, p = 0.009).

Conclusion

Different compositions of blood clots can show different TT2*RT and SI on MR imaging. Quantitative T2* mapping and multicontrast MR scanning can help in the characterization of clots causing large vessel occlusion, which is useful to establish treatment strategies for stroke patients.

## Introduction

Clot composition can impact revascularization outcomes and strategies in stroke patients with large vessel occlusion. In a preliminary study of 30 patients, a shorter thrombus-T2* relaxation time (TT2*RT) was found to be associated with earlier recanalization after thrombectomy using a combination of stent retriever and aspiration [1]. Other studies have suggested that clots with susceptibility vessel sign (SVS) on T2*-weighted gradient-echo (GRE) imaging are less likely to experience recanalization with intravenous tissue-type plasminogen activator [2]. Because of this potential to use magnetic resonance (MR) imaging for predicting clot composition, we performed an in vitro study to compare different MR imaging sequences for clot composition characterization, which might help to establish and individualize thrombectomy and thrombolytic strategies for patients with acute ischemic stroke.

## Materials and methods

Preparation of clot analogs

**Figure 1:**
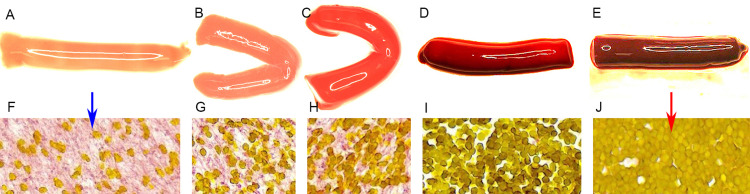
Histologic features of clots prepared using different RBC/fibrin ratios. A-E: Macrophotographs of clots with different ratios of RBC/fibrin (A, 5% RBC; B, 25% RBC; C, 50% RBC; D, 75% RBC; E, 95% RBC). The color of clots darkened as the RBC content increased from A to E. F-J: Microphotographs of the clots from A to E, showing more dense distribution of RBCs as the ratio of RBC/fibrin increased (from scattered RBCs [F, blue arrow] to densely arranged RBCs [J, red arrow]; MSB, original magnification 40×). RBC: red blood cell; MSB: Martius Scarlet Blue

Following Institutional Review Board approval from Mayo Clinic, whole blood was obtained from volunteers from the Blood Transfusion Service after quality control. Blood was separated into plasma, buffy coat, and erythrocyte-rich layers after centrifugation at 1,500 rounds per minute (RPM) for 12 minutes. The plasma and red blood cells (RBCs) were harvested and recombined in controlled ratios to prepare five different clot analogs (five analogs for each combination): Group A, fibrin-rich (95% plasma:5% RBCs); Group B, fibrin-rich (75% plasma:25% RBCs); Group C, intermediate (50% plasma:50% RBCs); Group D, RBC-rich (25% plasma:75% RBCs); and Group E, RBC-rich (5% plasma:95% RBCs). Clotting was initiated by adding 2.06% calcium chloride (CaCl_2_) solution in a 1:9 ratio (CaCl_2_ solution:blood mixture) followed by thrombin (10 IU/mL) (Sigma-Aldrich, St. Louis, MO, USA) in a 1:1,000 ratio (thrombin:blood mixture). The blood clot mixtures were quickly loaded into 3 mL plastic Luer-Lock syringes, which were spun at 20 RPM in a hybridization incubator for one hour at 37°C to prevent the natural sedimentation of RBCs in static conditions (Figure 1, Panels A-E) [3-5].

Phantom and magnetic resonance scans

To emulate the attenuation of the brain and skull, the clots were placed in a cylindrical phantom filled with 0.4 mM manganese chloride solution to produce MR image contrast similar to the vessel wall of the human carotid artery. For each clot type, five specimens were placed in the phantom. All 25 clots in the tank were then scanned simultaneously using a 3 Tesla MR imaging scanner (Signa Discovery^TM^ MR 750w, GE Healthcare, Waukesha, WI).

Quantitative T2* mapping and other MR sequences (T2 fast spin-echo [FSE], T2 GRE, fluid-attenuated inversion recovery [FLAIR], and susceptibility-weighted angiography [SWAN]) were used. Specific MR scanning parameters are summarized in Table 1. TT2*RT and MR signal intensities (SIs) were acquired on different images within the selected clot area on a GE HealthCare Advantage Workstation (GE Healthcare, Chicago, IL).

**Table 1:** A summary of the parameters of MR sequences. FOV: field of view; NEX: number of excitations; FSE: fast spin-echo; GRE: gradient-echo; FLAIR: fluid-attenuated inversion recovery; SWAN: susceptibility-weighted angiography

	TR/TE (ms)	TI (ms)	Flip angle (degrees)	Echo	Slice thickness (mm)	Slice gap (mm)	FOV (cm)	Matrix	NEX
T2* mapping	1,200/6.8		90	1/8	4	0.8	18 × 18	320 × 256	1.00
T2 FSE	3,634/96.8		111	1/1	3	0	18 × 18	512 × 512	4.00
T2 GRE	200/8.3		15	1/1	3	0	18 × 18	512 × 512	2.00
FLAIR	11,000/144.5	2,633.5	160	1/1	3.6	0	22 × 22	512 × 512	1.00
SWAN	74.6/24.2		15	1/1	1	-0.5	18 × 18	384 × 300	0.69

Histologic analysis

Clot analogs were analyzed histologically to confirm clot composition. Prepared clot analogs were fixed in 10% phosphate-buffered formalin for two days. Formalin-fixed specimens were embedded in paraffin and cut at a thickness of 3 µm. Representative slides from each analog were stained with Martius Scarlet Blue (MSB) on which RBC was stained as yellow and fibrin was stained as red (Figure 1, Panels F-J). MSB-stained slides were scanned and analyzed using Aperio ImageScope software (Leica Biosystems, Wetzlar, Germany).

Statistical analysis

Normality test of TT2*RT and SI was done using the Shapiro-Wilk test. Differences in SI between five groups (Groups A to E) on T2 mapping, T2 FSE, T2 GRE, FLAIR, and SWAN were compared using a one-way analysis of variance (ANOVA). Comparisons of differences in TT2*RT between five groups (Groups A to E) on T2 mapping were performed using the Mann-Whitney U-test. Correlation analysis between TT2*RT and SI values on T2 mapping was performed using the Pearson correlation test. Differences in SI between groups were considered significant if the p-value was less than 0.05.

## Results

Clot magnetic resonance features on T2* mapping

**Figure 2:**
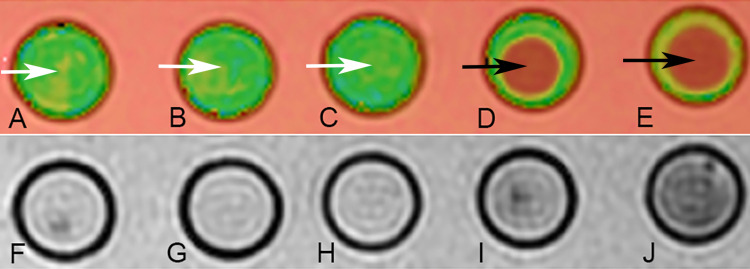
MR features from quantitative T2* mapping. Relaxation time (A-E) and SI values (F-J). As the RBC content increased, the average T2* relaxation time decreased from 126 to 37 ms, and the image color changed from green (white arrows) to red (black arrows) in the T2* estimation images (A to E). SI values dropped as the RBC content increased, and the image became darker (F to J). MR: magnetic resonance; SI: signal intensity; RBC: red blood cell

The average TT2*RT of the clots with lower RBC content (5%, 25%, and 50%) was 126.2 ± 21.3, 123.4 ± 19.9, and 118.8 ± 20.3 ms, respectively. The value for higher RBC content clots (75% and 95%) was 42.6 ± 1.4 and 37.0 ± 0.6 ms, respectively (Table 2). As the TT2*RT data did not show normal distribution, the Mann-Whitney U-test was chosen for further analysis. There were significant differences in TT2*RT between clots with lower and higher RBC content (p < 0.05 for all groups). Differences between groups with lower RBC content were not significant (p > 0.05). The TT2*RT from 75% RBC group was longer than 95% RBC group (p < 0.05). Overall, TT2*RT decreased from 126 to 37 ms from the most fibrin-rich clot to the most RBC-rich clot (about three times higher in the fibrin-rich group) (Figure 2, Panels A-E).

The mean SI value of clots with lower RBC content (5%, 25%, and 50%) on T2 mapping was 1,752 ± 71, 1,723 ± 28, and 1,700 ± 32, respectively. The SI value in clots with higher RBC content (75% and 95%) was 1,194 ± 106 and 842 ± 113, respectively (Table 2). As the normal distribution of the SI data was confirmed, one-way ANOVA was selected for further comparison. The difference in SI between clots with lower and higher RBC content was significant (P < 0.00001), which indicated the SI from T2 mapping reduced as the RBC content increased (Figure 2, Panels F-J). There was no significant difference in SI values between Groups A, B, and C (p > 0.05). The difference in SI between Groups D and E was significant (p = 0.0003). There was a strong positive correlation between TT2*RT and SI (R = 0.9628, p = 0.009).

**Table 2:** T2 mapping relaxation time and signal intensity in the five groups.

Groups	T2* relaxation time (ms)	Signal intensity
A	126.2 ± 21.3	1,752 ± 71
B	123.4 ± 19.9	1,723 ± 28
C	118.8 ± 20.3	1,700 ± 32
D	42.6 ± 1.4	1,194 ± 106
E	37.0 ± 0.6	842 ± 113

Signal intensity value changes in other magnetic resonance sequences

**Figure 3:**
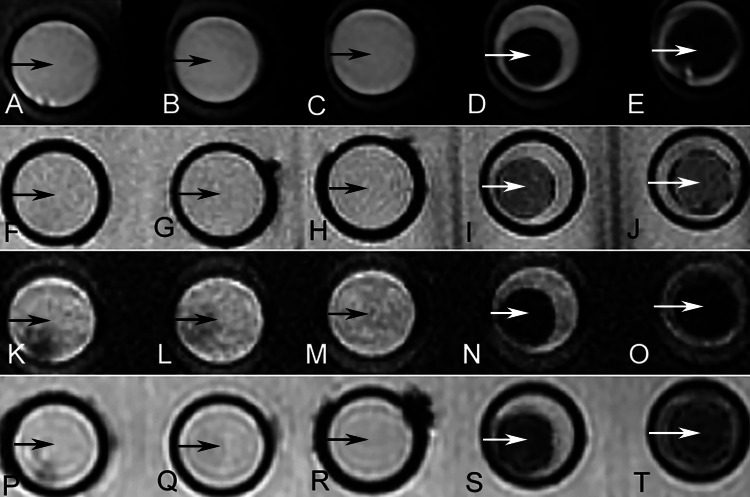
MR signals from other scanning sequences. Images on T2 FSE (from A [5% RBC] to E [95% RBC]), T2 GRE (from F [5% RBC] to J [95% RBC]), FLAIR (from K [5% RBC] to O [95% RBC]), and SWAN (from P [5% RBC] to T [95% RBC]). The lower signal is shown in clots with high RBC content (D, E, I, J, N, O, S, T, white arrows) by comparing with low RBC content (A, B, C, F, G, H, K, L, M, P, Q, R, black arrows). MR: magnetic resonance; FSE: fast spin-echo; RBC: red blood cell; GRE: gradient-echo; FLAIR: fluid-attenuated inversion recovery; SWAN: susceptibility-weighted angiography

Mean SI values of the clots from 5% to 95% RBC content were 3,264, 3,151, 3,075, 1,567, and 928 on T2 FSE imaging; 1,458, 1,411, 1,392, 953, and 875 on T2 GRE imaging; 1,832, 1,785, 1,778, 858, and 479 on FLAIR imaging; and 5,756, 5,518, 5,416, 2,586, and 1901 on SWAN imaging; respectively (Table 3). All the SI data fit the normal distribution, hence, one-way ANOVA was used for further comparison. The difference in SI between clots with lower (5-50%) and higher (75-95%) RBC content was significant on all comparisons (all p-values less than 0.00001), which indicated that the T2 FSE, T2 GRE, FLAIR, and SWAN SI values decreased as the RBC content increased (Figure 3, Panels A-T). The SI between groups with lower RBC content was not significantly different in all four sequences. The difference in SI between groups with higher RBC content (75% and 95%) was not significantly different in all sequences (p > 0.05) except on FLAIR (p = 0.0003).

**Table 3:** Signal intensities on other MR sequences. RBC: red blood cell; FSE: fast spin-echo; GRE: gradient-echo; FLAIR: fluid-attenuated inversion recovery; SWAN: susceptibility-weighted angiography; MR: magnetic resonance

		5% RBC	25% RBC		50% RBC		75% RBC		95% RBC	
T2 FSE		3,264 ± 54	3,151 ± 150		3,075 ± 104		1,567 ± 152		928 ± 120	
T2 GRE		1,458 ± 25	1,411 ± 62		1,392 ± 19		953 ± 192		875 ± 38	
FLAIR		1,832 ± 75	1,785 ± 120		1,778 ± 75		858 ± 80		479 ± 60	
SWAN		5,756 ± 163	5,518 ± 423		5,416 ± 248		2,586 ± 744		1,901 ± 112	

## Discussion

Our study demonstrated that the TT2*RT of clots decreased as the RBC content increased; hence, T2* mapping can provide more quantitative insight into clot composition. In addition, there was a positive correlation between TT2*RT and SI in the clots with different RBC content. Different MR SIs from different clots with varied RBC content was seen on various MRI sequences (T2 FSE, GRE, FLAIR, and SWAN). Hence, clot characterization can be performed using MR imaging to help guide and individualize both medical and endovascular therapy. An in vitro MR study of clot composition has been reported before [6]. However, our study is more comprehensive as we used multiple scanning sequences for the analysis.

As seen in our study, RBC-rich clots indicated localized hypointense signal at the site of the clot on all T2-weighted sequences. This might be caused by hemoglobin from RBCs known as susceptibility effects, which is related to the SVS [7,8]. There appears to be a consensus in the literature that the presence of an SVS is associated with improved revascularization outcomes and improved functional outcomes with mechanical thrombectomy [2,9,10]. In a study of 180 patients, the presence of the SVS was associated with 2.5 times higher odds of successful revascularization and significant improvements in functional outcomes [10]. In a quantitative study by Bourcier et al., the presence of the SVS was associated with a higher revascularization rate and more favorable clinical outcome with mechanical thrombectomy [11]. Regarding medical therapy, the presence of the SVS was reported to predict lower revascularization rates and poor clinical outcomes in acute stroke patients treated with intravenous tissue plasminogen activator alone [2]. Thus, SVS identification is both reliable and reproducible.

SWAN was reported to be more sensitive than T2*-weighted GRE and T2*-weighted GRE was more sensitive than T2 FSE sequences for the visualization of SVS in the intracranial arteries during the acute phase of ischemic stroke. SWAN is especially helpful to identify small clots [12-16]. In our study, RBC-rich clots were shown as low-signal from all T2-weighted images (irrespective of SWAN), possibly because our clots were not small. If the clot is small, we may need to perform SWAN for clot composition identification. In addition, FLAIR has been reported to show only hyperintense vessel sign in the occluded cerebral vessel [17], which is different from our study because both high (for fibrin-rich clot) and low (for RBC-rich clot) signal could be seen on our FLAIR images. In addition, there was a significant difference in SI between 75% and 95% RBC-rich clots using FLAIR, which is different from other T2-weighted MR sequences (no significant difference was seen between 75% and 95% RBC-rich clots). The underlying mechanisms for this are still unknown.

Hashimoto et al. [18] reported that the interaction between the stent-retriever struts and the clot is likely dependent on the RBC content of the clots. Significantly better patient outcomes can be achieved when the entire clot is removed on the first pass without losing any fragments. A fibrin-rich clot can adhere to the vessel wall and is difficult to dislodge using the stent retriever and/or the aspiration catheter; on the other hand, a weakly organized RBC-rich clot can be prone to fragmentation during the stent/catheter pull. Thrombi containing atheromatous gruel might be easily broken into smaller fragments than RBC-rich thrombi and reduce the effectiveness of thrombectomy. Thus an imaging screening protocol to provide an insight to the proportion of RBCs and fibrin in clots would be helpful for selecting a better treatment strategy.

This study has limitations because it was an in vitro study. There was no blood flow around the clots and the clot analogs were not in human cerebral vessels. We acknowledge these limitations of our study, and further investigation should be carried out to address this issue.

## Conclusions

Different TT2*RT and SI values from different compositions of blood clots can be seen on MR imaging. Quantitative T2* mapping and multicontrast MR scanning are useful for the characterization of clots causing large vessel occlusion, which helps to establish treatment strategies for stroke patients.
